# Early Healing Events after Periodontal Surgery: Observations on Soft Tissue Healing, Microcirculation, and Wound Fluid Cytokine Levels

**DOI:** 10.3390/ijms18020283

**Published:** 2017-01-27

**Authors:** Doğan Kaner, Mouaz Soudan, Han Zhao, Georg Gaßmann, Anna Schönhauser, Anton Friedmann

**Affiliations:** 1 Department of Periodontology, Witten/Herdecke University, 58455 Witten, Germany; mouaz.soudan@uni-wh.de (M.S.); anna-frederike.schoenhauser@uni-wh.de (A.S.); anton.friedmann@uni-wh.de (A.F.); 2 Multi-Disciplinary Treatment Center, Beijing Stomatological Hospital, Capital Medical University, Beijing 100050, China; z75301704@163.com; 3 praxisHochschule, University of applied sciences, 50670 Cologne, Germany; g.gassmann@praxishochschule.de

**Keywords:** periodontal surgery, periodontal regeneration, surgical crown lengthening, wound healing, cytokines

## Abstract

Early wound healing after periodontal surgery with or without enamel matrix derivative/biphasic calcium phosphate (EMD/BCP) was characterized in terms of soft tissue closure, changes of microcirculation, and expression of pro- and anti-inflammatory cytokines in gingival crevicular fluid/wound fluid (GCF/WF). Periodontal surgery was carried out in 30 patients (18 patients: application of EMD/BCP for regeneration of bony defects; 12 patients: surgical crown lengthening (SCL)). Healthy sites were observed as untreated controls. GCF/WF samples were collected during two post-surgical weeks. Flap microcirculation was measured using laser Doppler flowmetry (LDF). Soft tissue healing was evaluated after two weeks. GCF/WF levels of interleukin 1β (IL-1β), tumour necrosis factor (TNF-α), IL-6, and IL-10 were determined using a multiplex immunoassay. Surgery caused similar reductions of flap microcirculation followed by recovery within two weeks in both EMD/BCP and SCL groups. GCF/WF and pro-inflammatory cytokine levels were immediately increased after surgery, and returned only partially to baseline levels within the two-week observation period. Levels of IL-10 were temporarily reduced in all surgical sites. Flap dehiscence caused prolonged elevated levels of GCF/WF, IL-1β, and TNF-α. These findings show that periodontal surgery triggers an immediate inflammatory reaction corresponding to the early inflammatory phase of wound healing, and these inflammation measures are temporary in case of maintained closure of the flap. However, flap dehiscence causes prolonged inflammatory exudation from the periodontal wound. If the biological pre-conditions for periodontal wound healing are considered important for the clinical outcome, care should be taken to maintain primary closure of the flap.

## 1. Introduction

Periodontal surgery is routinely performed in order to remove microbial deposits from root surfaces and for corrective measures such as pocket elimination, removal of roots, or contouring of bony defects. Further, periodontal surgery allows placing of biomaterials or grafts for regeneration of lost periodontal structures [1]. Surgical lengthening of the clinical crown in order to increase retention of prospective restorations or to avoid subgingival restoration margins is another common periodontal surgical procedure [2].

Irrespective of the intention and the modalities of surgery, periodontal wound healing always begins with a blood clot in the space maintained by the closed flap after suturing [3]. In the early inflammation phase of wound healing, inflammatory cells are attracted by platelet and complement derived mediators and aggregate around the blood clot. While polymorphnuclear neutrophil granulocytes (PMN) dominate initially, monocytes and macrophages emerge within the first days [4]. The blood clot also provides a provisional matrix for cells originating from the surrounding tissues (i.e., gingiva, periodontal ligament (PDL), cementum, and alveolar bone) [5]. Thus, gingival fibroblasts, endothelial cells, osteoblasts, and special fibroblast populations originating from the PDL proliferate into the wound area. Wound healing progresses consequently through several phases from inflammation to cell proliferation and matrix formation and repair; then, these stages are followed by remodelling and maturation [4]. Reparative healing with formation of a long junctional epithelium and only little reorganization of connective tissue attachment is the expected outcome in periodontal wound healing without regenerative measures [6,7].

Clinical and human histological data show the efficacy of enamel matrix derivative (EMD) for inducement of periodontal regeneration [8,9,10]. These effects are mainly attributed to the proven stimulatory effects of EMD on PDL fibroblasts, cementoblasts, and osteoblasts [11,12,13], as well as to inhibitory effects on the competing epithelial cells [14]. However, other mechanisms may also contribute to the beneficial effects of EMD. Since a high microbial load negatively affects “gain” of clinical attachment after periodontal surgery [15], antibacterial effects of the EMD preparation may add to the stimulation of regeneration [16,17]. As wound healing is regulated by endogenous substances such as pro- and anti-inflammatory cytokines, the resolution of inflammation appears important for the outcome of wound healing [18]. For example, tissue destruction due to high levels of pro-inflammatory mediators like interleukin 1β (IL-1β), IL-6, and tumour necrosis factor (TNF-α) are reversed by the anti-inflammatory cytokine IL-10 [19]. Suppression of various pro-inflammatory cytokines and anti-inflammatory modulation of macrophages have been confirmed for EMD in vitro [20,21]. Direct influences have been shown on T helper lymphocyte migration, CD25 activation, and apoptosis in a three-dimensional collagen matrix migration model [22]. Correspondingly, these immunomodulatory effects of EMD may promote favourable conditions for periodontal wound healing.

Regardless of with or without application of bioactive substances, the course of early healing after periodontal surgery is scarcely investigated in humans, although the first post-surgical weeks are considered important [23]. The aim of this descriptive study was to characterize early wound healing after periodontal surgery with or without application of EMD combined with granular biphasic calcium phosphate (BCP) in terms of soft tissue closure, changes of microcirculation, and expression of pro- and anti-inflammatory cytokines.

## 2. Results

### 2.1. Patients

Thirty patients were recruited, treated, and analysed (EMD/BCP group: 18 patients; SCL group: 12 patients). Demographic and clinical characteristics are shown in Table 1.

### 2.2. Microcirculation

**Figure 1:**
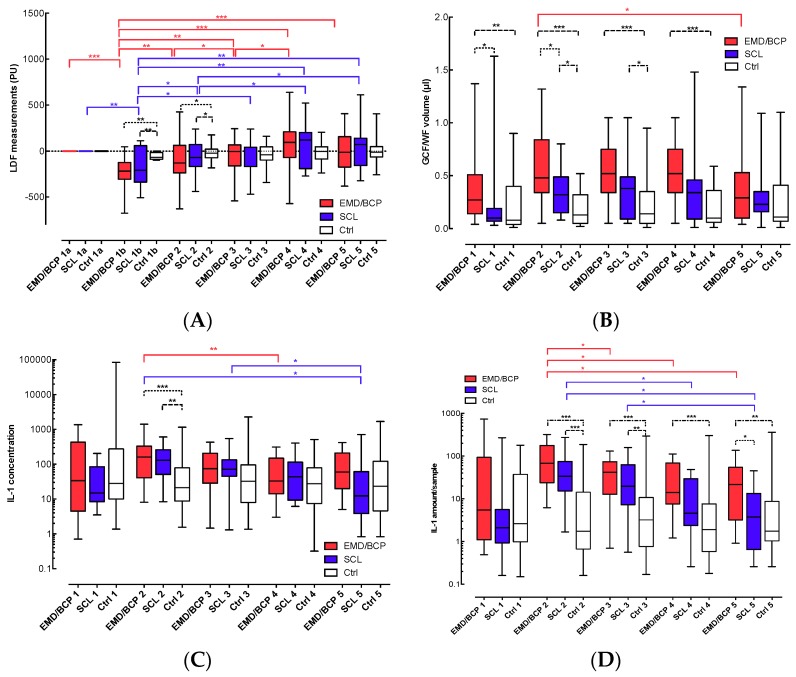
(**A**) LDF measurements of microcirculation; (**B**) Measurements of GCF/WF volume; (**C**) Concentration of IL-1β in GCF/WF samples; and (**D**) Total amount of IL-1β in GCF/WF samples. EMD/BCP (enamel matrix derivative/biphasic calcium phosphate): red, SCL (surgical crown lengthening): blue, Ctrl (control): white; LDF: laser Doppler flowmetry; GCF/WF: gingival crevicular fluid/wound fluid; IL-1: Interleukin 1β; *: *p* < 0.05; **: *p* < 0.01, ***: *p* < 0.001.

In each group, untreated control sites did not show any significant changes of perfusion throughout all 14 days of observation. Directly after surgery (D0b), both EMD/BCP and SCL groups showed significantly reduced microcirculation values at the papillary base compared to baseline at D0a (Figure 1A). Although perfusion increased significantly from termination of surgery (D0b) to D1 in both surgery groups, microcirculation was significantly lower than at untreated control sites not only after surgery (D0b), but also at D1. The ongoing significant recovery until 14 days after surgery (D14) was similar in both surgery groups.

### 2.3. GCF/WF

The results for analyses of GCF/WF samples and their changes are shown in Figure 1B. At baseline (D0a), GCF sample volume was significantly greater in EMD/BCP sites, when compared to SCL and control sites. One and three days after surgery (D1, D3), both surgical groups showed significantly higher sample volumes than found in control sites. In addition, WF sample volume at D1 was significantly greater in EMD/BCP sites than in SCL sites. Further, EMD/BCP sites showed higher volumes of WF at days three and seven (D3, D7), when compared to control sites.

### 2.4. Cytokine Levels at EMD/BCP, SCL, and Control Sites

All three groups showed similar concentrations of IL-1β at baseline (Figure 1C). One day after surgery (D1), IL-1β concentrations were similarly elevated over healthy control sites in both surgical groups (EMD/BCP: 7.5-fold, SCL: 6-fold; *p* < 0.05). In both EMD/BCP and SCL sites, the concentrations of IL-1β decreased significantly again until the end of the observation period (from D3 to D7).

At baseline (D0a), all three groups showed similar amounts of IL-1β/sample (Figure 1D). At day one (D1) and at day three (D3), the amounts of IL-1β were significantly enhanced in EMD/BCP and SCL samples, when compared to control samples (39-fold and 19-fold, respectively, *p* < 0.001). The amount of IL-1β/sample decreased significantly for both surgical groups from D1 to D14. However, IL-1β levels detected in EMD/BCP sites after 14 days (D14) were still significantly elevated, when compared to both other groups (6-fold and 12-fold, respectively, *p* < 0.05).

**Figure 2:**
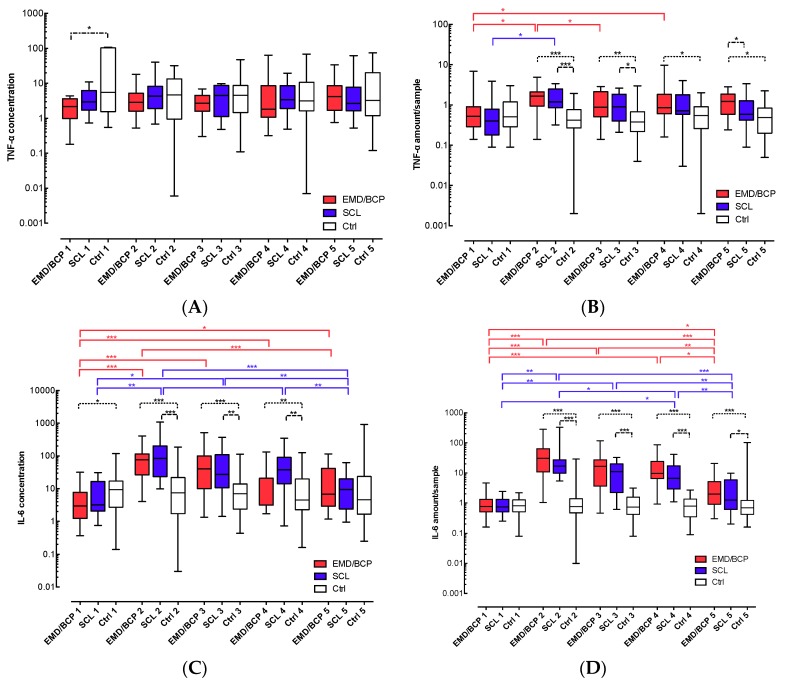

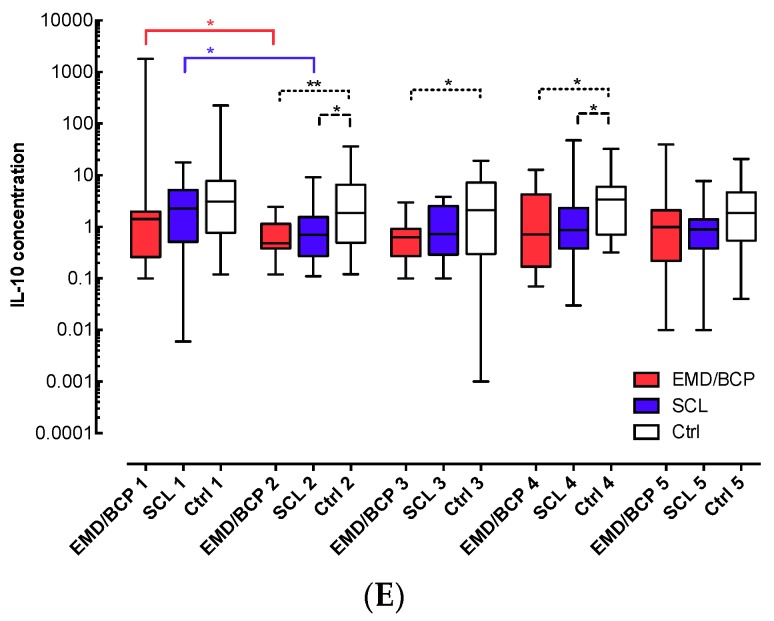
(**A**) Concentration of TNF-α in GCF/WF samples; (**B**) Total amount of TNF-α in GCF/WF samples; (**C**) Concentration of IL-6 in GCF/WF samples; (**D**) Total amount of IL-6 in GCF/WF samples; (**E)** Concentration of IL-10 in GCF/WF samples. EMD/BCP (enamel matrix derivative/biphasic calcium phosphate): red, SCL (surgical crown lengthening): blue, Ctrl (control): white; GCF/WF: gingival crevicular fluid/wound fluid; TNF-α: Tumour necrosis factor α; IL-6: Interleukin 6; IL-10: Interleukin 10; *: *p* < 0.05; **: *p* < 0.01, ***: *p* < 0.001.

At baseline (D0a), control sites showed a significantly higher concentration of TNF-α than EMD/BCP sites. No further differences or significant changes were found (Figure 2A).

All three groups showed similar amounts of TNF-α/sample prior to surgery at D0a (Figure 2B). After one and three days (D1, D3), the amount of TNF-α had significantly increased in both surgical groups and exceeded the levels found in control sites significantly. In the EMD/BCP group, the amount of TNF-α/sample remained high, and a significantly greater amount of TNF-α was found in EMD/BCP sites after 14 days (D14) in comparison to both SCL and healthy sites (2- and 2.5-fold, respectively, *p* < 0.05).

Prior to surgery (D0a), the concentration of IL-6 in EMD/BCP sites was significantly lower than in control sites (Figure 2C). In both surgical groups, the concentration of IL-6 increased significantly after surgery, and decreased again from D1 to D7. Accordingly, the EMD/BCP and SCL groups showed significantly higher IL-6 concentrations than controls at D1, D3, and D7.

The amounts of IL-6/sample were similar in all groups at baseline (D0a) (Figure 2D). At D1, the amounts of IL-6 were significantly increased by a factor of 39 for EMD/BCP and 22 for SCL, respectively (*p* < 0.001), and decreased significantly again over the course of 14 days (D3 to D14, *p* < 0.05).

However, when compared to controls, both surgical groups showed significantly higher amounts of IL-6 continuously from D1 to D14 (*p* < 0.05).

The concentration of IL-10 decreased similarly and significantly between D0a and D1 in both EMD/BCP and SCL groups; accordingly, significantly reduced concentrations of IL-10 were found in both surgical groups at D1, D3, and at D7, when compared to healthy sites (0.25- to 0.4-fold in both surgery groups, *p* < 0.05; Figure 2E).

The amount of IL-10/sample did not change significantly over time within all groups, and significant differences among the groups were not detected (data not shown).

### 2.5. Soft Tissue Healing

**Figure 3:**
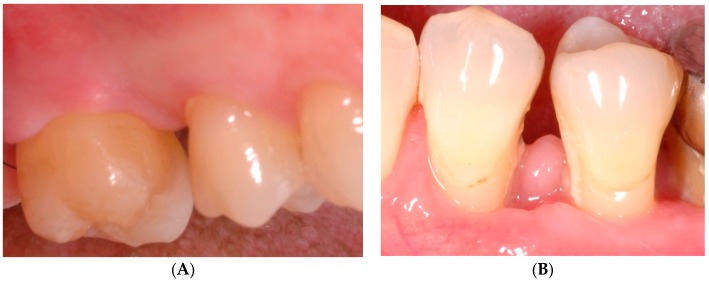
Soft tissue healing two weeks after surgery (EMD/BCP group): (**A**) EHI 1 and closed flap; (**B**) EHI 5 and flap dehiscence. EMD/BCP: enamel matrix derivative/biphasic calcium phosphate; EHI: early healing index.

Two weeks after surgery (D14), the SCL group presented significantly better EHI values compared to the EMD/BCP group (*p* = 0.032, Table 2, Figure 3). While 13 EMD/BCP sites presented inter-proximal dehiscence of the flap, only three sites with dehiscence were observed in SCL patients (*p* = 0.024, Table 3, Figure 3).

### 2.6. GCF/WF/Cytokine Levels at Sites with or without Flap Dehiscence

**Figure 4:**
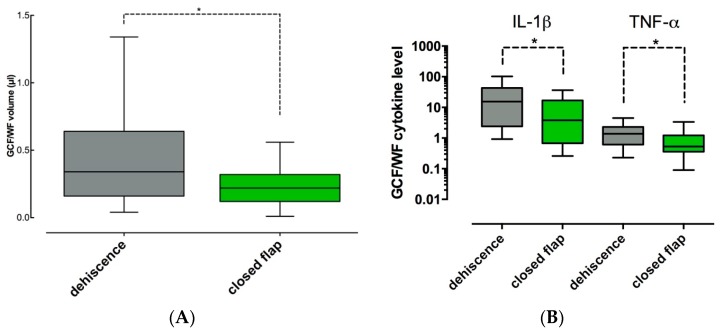
GCF/WF and cytokine levels in sites with/without dehiscence (pooled results for all sites/both groups): (**A**) Significantly higher GCF/WF volume were found in sites with flap dehiscence, when compared to closed flaps (*: *p* < 0.05); (**B**) Significantly higher total amounts/sample of IL-1β and TNF-α were found in sites with flap dehiscence, when compared to closed flaps (*: *p* < 0.05). Dehiscence: grey, closed flap: green.

Sixteen of thirty surgical sites displayed dehiscent inter-proximal flaps after two weeks, when the results for all sites of both groups were pooled. In these sites, the amount of GCF/WF was 1.5-fold elevated (*p* = 0.049, Figure 4A). In GCF/WF of sites with dehiscence, the total amounts/sample of IL-1β and of TNF-α were 4-fold and 2.5-fold increased, when compared to closed sites (*p* = 0.033 and *p* = 0.021, Figure 4B). Other cytokine parameters were unaffected by the course of soft tissue healing (*p* > 0.05, data not shown).

## 3. Discussion

The main events of periodontal wound healing are completed within two to three weeks of wound closure, followed by tissue maturation and remodelling [4]. The aim of this descriptive study was to characterize this early phase of wound healing in terms of soft tissue closure, changes of microcirculation, and expression of pro- and anti-inflammatory cytokines.

Maintenance of inter-proximal flap closure was assessed using both the Early Healing Index (EHI) and a dichotomous classification (dehiscence yes/no). EHI values ranging from 2–3 (complete flap closure with presence of a fibrin line or fibrin clot) were found in 75% of patients treated with SCL, while 25% of SCL sites showed EHI values of 4–5 (incomplete flap closure with partial or complete inter-proximal necrosis), or dehiscence of the flap. Suchlike inter-proximal flap dehiscences despite initial primary closure occur frequently after resective periodontal surgeries and heal by second intention [24]. Healing by second intention causes formation of gingival clefts and craters, but these soft tissue deformities may even out over time and are of limited clinical relevance after resective surgery [25].

Significantly greater proportions of dehiscent flaps were found in EMD/BCP patients (72%), which is an unexpected finding. The frequency of incomplete flap closure ranged from 0%–10% in similar studies combining minimally invasive techniques with EMD [26,27,28], and therefore, the high proportion of inter-proximal dehiscence is surprising. Generally, local anaesthesia and flap elevation disturb microcirculation and induce ischemia [29], while maintenance of blood flow is essential for survival of the operated tissue [30]. In our study, the effects of surgery on flap perfusion were evaluated using Laser Doppler flowmetry (LDF). LDF measurements of microcirculation detect disturbances in blood flow caused by surgical trauma and are able to discriminate surgical techniques according to different extents of tissue traumatisation [31,32]. Further, LDF has been shown to predict flap dehiscence after surgery [32]. Both groups showed significant reductions of microcirculation directly after surgery and after one day, when compared to untreated control sites. However, microcirculation returned quickly to unimpaired levels, and no significant difference with regard to reduction of blood flow was found between EMD/BCP and SCL sites. Since suchlike alterations of microcirculation are normal responses to surgery, the poor outcome of soft tissue healing in the EMD/BCP group should not be attributed to insufficient blood flow caused by allegedly excessive tissue traumatisation during treatment. Instead, it should be scrutinised that the surgeon attempted to re-establish the normal convex inter-proximal bony contour by adding grafting material supra-crestally beyond the remaining margins of the bone defect. The ideal amount of granular materials for periodontal defects is not known. Enhancement of flap support at non-containing defects is an important rationale for implantation of defect fillers; however, the placement of bone substitutes and the selection of graft type is considered subordinate to primary closure and wound stability [33]. In our study, overfilling of the bony defects may have promoted flap dehiscence in EMD sites, especially since coronal advancement of the flaps had not been carried out. Consequently, clinicians applying bone substitutes during regenerative periodontal surgery should always consider resultant difficulties in maintaining primary closure of the flap.

Irrespective of the treatment applied, surgery resulted in elevated volumes of GCF/WF samples and the levels of IL-1β, TNF-α, and IL-6 were markedly increased as early as after one day. The early peak of these pro-inflammatory cytokines relates to the physiologic reaction to tissue injury, the early inflammatory phase of wound healing [34]: IL-1β and TNF-α are necessary for inducement of expression of adhesion molecules and chemokines, secretion of other inflammatory mediators, and of matrix metalloproteinases [35]. Lack of these pro-inflammatory cytokines in the early phase, however, delays or even impairs wound healing [36,37,38]. After the initial peak, an incremental reduction of the amount of GCF/WF and the levels of IL-1β and TNF-α was found at most sites during 14 days, which may correspond to the transition between different phases of wound healing [34]. In contrast to IL-1β and TNF-α, the elevated levels of IL-6 were maintained over the entire observation period of 14 days. Levels of IL-6 are generally elevated during the early phase of cutaneous wound healing, since IL-6 has been shown to be involved in regulation of leukocyte infiltration and angiogenesis [37,39]. Unlike IL-1β, TNF-α, and IL-6, IL-10 is an immunosuppressive cytokine and regulates innate and adaptive immune responses [34]. Both surgical groups showed significant reductions of IL-10 concentrations in GCF/WF one day after surgery and the reduced levels were maintained for one week. Considering the contrasting levels of IL-1β and TNF-α, the decreased levels of Il-10 may truly reflect the first inflammatory phase of wound healing [34].

Given the well-known anti-inflammatory effects of EMD found in vitro, reduced levels of pro-inflammatory cytokines or enhanced levels of IL-10 were to be expected in GCF/WF samples of EMD/BCP sites. Interestingly, we failed to reproduce the effects of EMD found in vitro in our clinical study. This is in line with a recent report by Villa et al., who found a similar discrepancy between cytokine levels in clinical samples of wound fluid after periodontal surgery and in vitro measurements [40]. However, in contrast to “clean” in vitro assays focusing on one particular cell type such as PDL fibroblasts, osteoblasts, or keratinocytes, a wide variety of different cell types and exudates will always contribute to GCF/WF samples, which may obscure the detectable effects of biological mediators on a given cell population within the periodontal wound. Further, in the study by Villa et al. [40], conventional macro-surgical flap designs and suture materials were used instead of micro-surgical or minimally invasive techniques and materials, which may have negatively affected the parameters assessed for characterization of early wound healing. Similarly, the poor outcome of soft tissue healing in EMD/BCP sites in our study may have contributed to the lack of detectable effects of EMD on cytokine levels. Inflammation, as characterized by high levels of IL-1β, reduces the beneficial effects of EMD on wound healing: wound fill rate, cell proliferation and adhesion, synthesis of growth factors and collagen as well as mineralization were negatively affected under concomitant challenge with IL-1β in vitro [41]. In our study, a suchlike interrelation between inflammation and scarce effect of EMD may be clinically reflected by the fact that significantly higher levels of GCF/WF, IL-1β, and TNF-α were found in sites with inter-proximal flap dehiscence, despite application of EMD. Flap dehiscence always leads to bacterial colonization of the surgical site and especially of implanted materials [42,43]. Subsequently, the early physiologic inflammatory response to wounding cannot be resolved and becomes chronic due to the bacterial infection, with detrimental effects on the attempted regeneration [44]. For example, prolonged high levels of IL-1β and TNF-α impair wound healing by inhibition of collagen synthesis [45,46]. In contrast, inflammation at non-infected sites with maintained flap closure can be reduced promptly by pro-resolving mediators, and normal healing can occur [44].

In conclusion, periodontal surgery immediately increased the amount of GCF/WF and the levels of pro-inflammatory cytokines, which was reflected by contrasting effects on the anti-inflammatory cytokine IL-10. Increased measures of inflammation were temporary in case of maintained closure of the flap. However, flap dehiscence caused prolonged inflammatory exudation from the periodontal wound. If the biological pre-conditions for periodontal wound healing are considered important for the clinical outcome, care should be taken to maintain primary closure of the flap.

## 4. Materials and Methods

### 4.1. Treatment Groups

The study protocol was approved by the institutional Ethics Committee of the Witten/Herdecke University (no. 39/2011; 24 April 2011). The study was conducted in accordance with the guidelines of Good Clinical Practice (GCP-ICH) and the principles of the Helsinki Declaration of 1975, as revised in 2008. Written informed consent was obtained from all patients. Two groups of patients were treated with periodontal surgery and monitored during the first two weeks of healing:

(1) regenerative periodontal surgery of intrabony defects, using enamel matrix derivative and a granular bone substitute (EMD/BCP group).
(2) surgical crown lengthening prior to prosthetic treatment (SCL group).

### 4.2. Study Patients

Participants were recruited from the patient pool of the Witten/Herdecke University’s Dental School (first patient in: 17 May 2011; last patient out: 27 September 2012). Patients presenting a periodontal site in need of regenerative therapy (probing depth (PD) ≥ 6mm, and radiographic evidence of a vertical bone defect of at least 4 mm, assessed at a supportive periodontal therapy visit three months after completion of non-surgical therapy for moderate to advanced generalized chronic periodontitis) were assigned to the EMD/BCP group. A minimal width of 2 mm of keratinized gingiva at the prospective surgical site and an inter-proximal control site considered “clinically healthy” (PD ≤ 3 mm, negative for bleeding on probing and suppuration) were also required.

Periodontally healthy patients (no site with PD > 3 mm, full-mouth bleeding on probing score <25%, mean full-mouth plaque score <25%) were recruited to the SCL group in case of presence of a site in need of surgical crown lengthening for prosthetic reasons (distance of a planned restoration margin to bone crest ≤2 mm). A minimal width of 2 mm of keratinized gingiva at the prospective surgical site and presence of a control site considered “clinically healthy” (PD ≤ 3 mm, negative for bleeding on probing and suppuration) were also required.

Exclusion criteria included pregnancy, lactation period, use of antibiotics or anti-inflammatory drugs in the previous six months, systemic diseases and medications affecting periodontal inflammation or surgical procedures, and any condition requiring premedication before dental treatment.

### 4.3. Surgery

All surgeries were carried out by the same experienced surgeon (GG). Patients were advised to abstain from brushing the surgical area, and to use a 0.2% chlorhexidine mouth rinse twice daily for 1 min until removal of sutures.

#### 4.3.1. Access Flap with EMD and Granular Bone Substitute (EMD/BCP)

**Figure 5:**
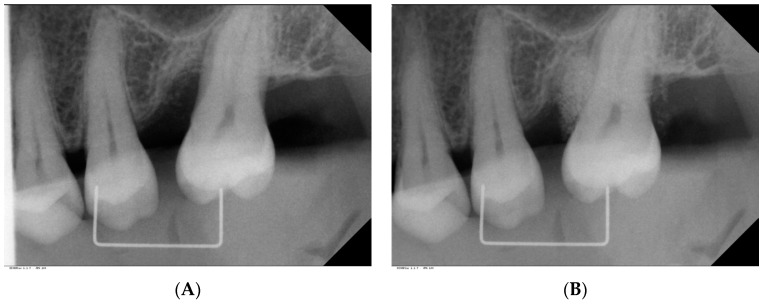
Pre- and post-surgical radiographs (EMD/BCP group). (**A**) Deep vertical infra-bony defect is visible on the mesial aspect of tooth 27; (**B**) The bone defect is filled with radio-opaque bone substitute (BCP) beyond the residual contour of the alveolar crest.

The simplified papilla preservation flap technique (SPPF) [47] was used in case of an interdental width of ≤2 mm, as measured at the papilla base. A modified papilla preservation flap (MPPF) [48] was performed at sites presenting with an interdental width of >2 mm. Vertical releasing incisions were avoided. After flap elevation, thorough debridement of the root surface, and degranulation, the defect was rinsed with sterile saline. A 24% ethylenediamine tetraacetic acid gel (PrefGel, Institut Straumann AG, Basel, Switzerland) was applied to the exposed root surface and removed after 2 min using saline. Then, EMD (Emdogain, Institut Straumann AG, Basel, Switzerland) was administered to the root surface. The bone defect was filled with a granular bone substitute (biphasic calcium phosphate (BCP); Bone Ceramic, Institut Straumann AG, Basel, Switzerland) mixed with EMD, using a sterile amalgam gun. BCP was not only applied into the bone defect for flap support, but also used for vertical augmentation beyond the remaining defect margins in order to compensate for both vertical and horizontal bone loss and to re-establish the physiologic convex inter-proximal bone contour (Figure 5A,B). Then, the flaps were closed with modified vertical mattress sutures, using fine monofilament suture material (6.0). Sutures were removed after two weeks.

#### 4.3.2. Surgical Crown Lengthening (SCL)

After placement of bevelled internal and sulcular incisions, a mucoperiosteal flap was reflected and the bone was exposed. Vertical releasing incisions were avoided. Ostectomy and osteoplasty were carried out with burs and hand instruments in order to establish a distance of up to 3 mm from the projected restoration margin to the new bony crest, as described previously [2,49]. Then, the flaps were closed with vertical mattress sutures with periosteal anchorage for apical repositioning, using fine monofilament suture material (6.0). Sutures were removed after two weeks.

### 4.4. Assessment of Microcirculation

A laser Doppler flowmeter (Periflux 5010, Perimed AB, Jarfalla, Sweden) equipped with a PF 416 probe (outside diameter 1.0 mm, fibre separation 0.25 mm; wavelength 780 nm) was used for assessing changes of microcirculation at surgical and control sites before surgery (D0a), directly after completion of surgery (D0b), and one (D1), three (D3), seven (D7), and 14 days (D14) after surgery. The LDF probe was aligned at a custom-made acrylic stent and the measurements were carried out always at the same position (base of the buccal papilla) for 1 min, perpendicular to the tissue and at a distance of 0.5 mm to the flap. The flowmeter recordings were monitored using the Perisoft software (Perisoft 2.10, Perimed AB, Jarfalla, Sweden), measured in perfusion units (PU), and analysed as changes relative to the baseline value defined as zero.

### 4.5. Sampling of Gingival Crevicular Fluid/Wound Fluid (GCF/WF)

Samples of gingival crevicular fluid or wound fluid (GCF/WF) were harvested at one surgical and one healthy control site/patient before surgery (D0a), and after one (D1), three (D3), seven (D7), and 14 days (D14). For sampling, sites were isolated with cotton rolls and gently air-dried. A paper strip (Perio paper, Oraflow, Amityville, NY, USA) was inserted into the site until mild resistance was felt and left in place for 30 s. The GCF/WF sample volume was immediately determined with a micro-moisture meter (Periotron 8000, Oraflow, Amityville, NY, USA) and calculated in microliters from a standard curve. Samples were stored at −80 °C in dry vials until further processing.

### 4.6. Determination of GCF/WF Cytokine Levels

The GCF/WF levels of cytokines (IL-1β, TNF-α, IL-6, IL-10) were determined with a multiplex immunoassay (Magpix, Luminex, Austin, TX, USA) following the manufacturer’s instructions. The samples were thawed in assay buffer, shaken for 1 min with a vortex mixer, and centrifuged for 5 min at 3000× *g* for recovery of GCF. Data are presented in pg/mL for concentration and in pg/sample for total amount of cytokine per sample.

### 4.7. Assessment of Clinical Soft Tissue Healing

Pictures were taken from all surgical sites two weeks after surgery (D14). Using the photographs, wound closure was independently evaluated by two calibrated examiners blinded to the group allocation, using the Early Healing Index (EHI; 1: complete flap closure—no fibrin line in the inter-proximal area; 2: complete flap closure—fine fibrin line in the inter-proximal area; 3: complete flap closure—fibrin clot in the inter-proximal area; 4: incomplete flap closure—partial necrosis of the inter-proximal tissue; 5: incomplete flap closure—complete necrosis of the inter-proximal tissue) [28]; in addition, wound closure was assessed dichotomously (soft tissue dehiscence yes/no).

### 4.8. Statistical Analysis

Statistics were calculated with the patient as the unit of analysis (split-mouth design, using one surgical site and one control site/patient). Non-parametric statistical tests were used according to the non-normal data distribution (confirmed with Kolmogorov-Smirnov test, data not shown). Medians and inter-quartile ranges (IQ) were calculated for metric parameters (LDF, GCF/WF volume, cytokine concentration, and total amount/sample). Wilcoxon’s signed-rank test was used for longitudinal comparisons of repeated measurements within the groups. Cross-sectional analyses were carried out with the Mann–Whitney *U* test. Categorical data were analysed using Pearson’s *Chi*^2^ test. A statistical software program was used for all calculations (SPSS 22 for OSX, SPSS Inc., Chicago, IL, USA). Statistical significance was defined as *p* < 0.05.

## Figures and Tables

**Table 1:** Demographic and clinical patient characteristics at baseline. EMD/BCP: enamel matrix derivative/biphasic calcium phosphate; SCL: surgical crown lengthening. IQ: inter-quartiles. n.d.: Not Detected.

Patient Data	EMD/BCP 18 Patients	SCL 12 Patients	*p*
Age (years, median, IQ)	58 (47, 71)	51 (48, 58)	>0.05
Gender (male/female)	9/9	7/5	>0.05
Cigarette smokers (yes/no)	3/15	1/11	>0.05
Probing depth (mm, median, IQ) Min/max	7 (6.3, 9) 6, 11	n.d.	-
median tooth mobility (0–III) Min/max	0 (0, 1) 0, 2	0	>0.05
Radiographic defect depth (mm, median, IQ) Min/max	6 (5, 7) 3, 9	0	<0.001

**Table 2:** Contingency table for wound healing in both groups, evaluated using the Early Healing Index (EHI). Significant difference favouring the SCL group (*p* = 0.032, Pearson’s *Chi*^2^ test). EMD/BCP: enamel matrix derivative/biphasic calcium phosphate; SCL: surgical crown lengthening.

EHI	1	2	3	4	5
EMD/BCP	2 (11%)	2 (11%)	1 (6%)	4 (22%)	9 (50%)
SCL	0 (0%)	5 (42%)	4 (33%)	1 (8%)	2 (17%)

**Table 3:** Contingency table for flap dehiscences (yes/no) noted two weeks after surgery. Significant difference favouring the SCL group (*p* = 0.024, Pearson’s *Chi*^2^ test). EMD/BCP: enamel matrix derivative/biphasic calcium phosphate; SCL: surgical crown lengthening.

	EMD/BCP	SCL
flap dehiscence	13 (72%)	3 (25%)
no dehiscence	5 (28%)	9 (75%)
